# *HNF1A *G319S variant, active cigarette smoking and incident type 2 diabetes in Aboriginal Canadians: a population-based epidemiological study

**DOI:** 10.1186/1471-2350-12-1

**Published:** 2011-01-05

**Authors:** Sylvia H Ley, Robert A Hegele, Stewart B Harris, Mary Mamakeesick, Henian Cao, Philip W Connelly, Joel Gittelsohn, Ravi Retnakaran, Bernard Zinman, Anthony J Hanley

**Affiliations:** 1 Department of Nutritional Sciences, University of Toronto, Toronto, ON, Canada; 2 Robarts Research Institute and University of Western Ontario, London, ON, Canada; 3 Center for Studies in Family Medicine, Schulich School of Medicine and Dentistry, University of Western Ontario, London, ON, Canada; 4 Sandy Lake Health and Diabetes Project, Sandy Lake, ON, Canada; 5 Department of Laboratory Medicine and Pathobiology, University of Toronto, Toronto, ON, Canada; 6 The Keenan Research Centre of the Li Ka Shing Knowledge Institute, St Michael's Hospital, Toronto, ON, Canada; 7 Center for Human Nutrition, Johns Hopkins Bloomberg School of Public Health, Baltimore, MD, USA; 8 Division of Endocrinology, University of Toronto, Toronto, ON, Canada; 9 Leadership Sinai Centre for Diabetes, Mount Sinai Hospital, Toronto, ON, Canada; 10 Samuel Lunenfeld Research Institute, Mount Sinai Hospital, Toronto, ON, Canada

## Abstract

**Background:**

In a recent report of large-scale association analysis, a type 2 diabetes susceptibility locus near *HNF1A *was identified in predominantly European descent populations. A population-specific G319S polymorphism in *HNF1A *was previously identified in Aboriginal Canadians who have a high prevalence of type 2 diabetes. We aimed to investigate the association of the *HNF1A *G319S polymorphism with incident type 2 diabetes and to assess whether clinical risk variables for type 2 diabetes influence the association in an Aboriginal population.

**Methods:**

Of 606 participants who were free of diabetes at baseline in 1993-1995, 540 (89.1%) participated in 10-year follow-up assessments in 2003-2005. Fasting glucose and a 75-g oral glucose tolerance test were obtained to determine incident type 2 diabetes. Participants were genotyped for the *HNF1A *G319S polymorphism. Interviewers administered questionnaires on smoking behavior.

**Results:**

The incidence rates of type 2 diabetes were 14.2% (55/388) in major allele homozygotes and 31.2% (29/93) in minor allele carriers (p < 0.001). The *HNF1A *G319S carrier status was associated with incident type 2 diabetes (odds ratio [OR] 3.78 [95% CI 2.13-6.69]) after adjustment for age, sex, hypertension, triglyceride, HDL cholesterol, and waist circumference. A statistical interaction was observed between *HNF1A *G319S and baseline active cigarette smoking on the development of type 2 diabetes with similar adjustment (p = 0.006). When participants were stratified by baseline smoking status, *HNF1A *G319S carriers who were active smokers had increased risk of developing diabetes (OR 6.91 [95% CI 3.38-14.12]), while the association was attenuated to non-significance among non-smokers (1.11 [0.40-3.08]).

**Conclusions:**

The *HNF1A *G319S variant is associated with incident type 2 diabetes in Aboriginal Canadians. Furthermore, cigarette smoking appears to amplify incident diabetes risk in carriers of *HNF1A *G319S.

## Background

Type 2 diabetes has become a global epidemic, particularly among Aboriginal Canadians who have a 3-5 times higher prevalence of the disease compared to non-Aboriginal Canadians [1]. Both environmental and genetic factors have been attributed to this diabetes epidemic. Aboriginal populations have been exposed to a rapid epidemiological transition in conjunction with unique genetic susceptibility to diabetes [2]. We have previously reported that a glycine to serine substitution at codon 319 (G319S) of the *HNF1A *gene in an Aboriginal population was significantly associated with increased type 2 diabetes prevalence in cross-sectional analysis [3].

The *HNF1A *gene codes for a transcription factor, hepatic nuclear factor (HNF)-1α, which plays an important role in pancreatic β-cell function [4-6]. In various populations, mutations in the *HNF1A *gene are a common cause of the maturity-onset diabetes of the young (MODY) [5-8], which is characterized by early age of onset and a marked defect in insulin secretion. Unlike these common *HNF1A *variants, the diabetes phenotype that emerged in S319 allele carriers was non-MODY type 2 diabetes [3]. The S319 allele was associated with accelerated onset of type 2 diabetes in a gene-dosage manner [9], with ~7 years earlier onset for each copy of the S319 allele carried.

In a recent report of large-scale association analysis, a type 2 diabetes susceptibility locus near *HNF1A *was identified in predominantly European descent populations [10]. However, there has been no prospective study investigating whether the population-specific variant, G319S, influences the development of type 2 diabetes in Aboriginal Canadians who have a high prevalence of type 2 diabetes [11]. Accompanying this high prevalence of type 2 diabetes, the population has high prevalence rates of diabetes risk variables including obesity and smoking [11-14], which are important public health concern. However, no study has assessed whether clinically measured risk variables influence the association between the G319S variant and incident type 2 diabetes in Aboriginal Canadians. We aimed to determine the association of the *HNF1A *G319S variant with the incidence of type 2 diabetes and to assess whether clinical risk variables for type 2 diabetes influence this association.

## Methods

### Study Population

The Sandy Lake Health and Diabetes Project is a population-based cohort study designed to determine the incidence of diabetes and its associated risk factors in an Aboriginal Canadian population. Between 1993 and 1995, baseline data were obtained from 728 of 1018 (72%) eligible residents of Sandy Lake First Nation aged 10-79 years [11]. Signed and informed consent was obtained from all participants, and the study was approved by the Sandy Lake First Nation Band Council and University of Toronto Ethics Review Committee.

Between 2003 and 2005, among 606 participants who were free of diabetes at baseline, 540 (89.1%) participated in the 10-year follow-up evaluation [14]. Those who did not return for follow-up (n = 66) were slightly younger compared with respondents, but not different according to sex and body mass index (BMI). After excluding participants who died during follow-up, who had missing baseline fasting and 2-hour postload glucose, or who had diabetes at baseline defined by the revised World Health Organization diagnostic criteria [15], 492 men and women remained in the present study.

### Baseline data collection and laboratory procedures

At baseline, blood samples were collected after an 8- to 12-hour overnight fast to determine fasting glucose, insulin, and lipid profile [11]. A 75-g OGTT was administered, and a second blood sample for glucose was drawn at 120 minutes postload. Glucose concentration was determined using the glucose oxidase method. Fasting plasma insulin concentration was analyzed by radioimmunoassay (Pharmacia, Piscataway, NJ). The homoeostasis model assessment (HOMA) of insulin resistance was estimated by the method of Matthews et al. [16]. Triglyceride, high-density lipoprotein (HDL) and low-density lipoprotein (LDL) cholesterol were determined using standard methods described in the Lipid Research Clinics manual of operations [17].

Each anthropometric/blood pressure measurement was performed twice, and the average was used in analyses. Height and weight were measured using an Accustat wall-mounted stadiometer (Genentech Inc., San Francisco, California) and a hospital balance beam scale (Health-o-Meter Inc., Bridgeview, Illinois), respectively. Waist circumference was measured at the iliac crest using an inelastic tape.

Interviewer-administered questionnaires were used to collect information on cigarette smoking behavior. Active smokers were defined as those reporting current use of cigarettes at the time of the baseline survey. These participants were asked about their average number of cigarettes smoked daily. Smoking pack-years were calculated by dividing the number of daily cigarettes used by 20 and then multiplying by the smoking duration up to baseline data collection.

### Genetic analyses

Restriction analysis with *Bse*DI followed by polyacrylamide gel electrophoresis was used to detect the DNA change underlying the *HNF1A *G319S amino acid variant [3]. DNA sequence-proven controls were run as standards for each genotyping reaction and a random 15% of samples were studied on another day with independent genotyping reactions. The concordance between replicates was 100%.

### Definition of incident type 2 diabetes

Incident type 2 diabetes at follow-up was defined as the presence of any one of the following at follow-up assessments: 1) fasting plasma glucose ≥ 7.0 mmol/l or 2-hour postload plasma glucose ≥ 11.1 mmol/l on a 2-hour OGTT; 2) current use of insulin or oral hypoglycemic agents; or 3) a positive response to the question: Have you ever been diagnosed with diabetes by a nurse (practitioner) or a doctor [14]? Of 492 participants, follow-up blood samples were collected from 383 (77.8%) participants, while the diabetes status of 109 (22.2%) participants, who were not available for the follow-up blood sampling visit, was ascertained based on self-reported clinical diagnosis of diabetes through a phone interview. The distributions of S319 allele carriers between those with and without blood samples at follow-up were not statistically different (p = 0.85).

### Statistical analysis

Distributions of continuous variables were assessed for normality, and natural log transformations of skewed variables were used in statistical analyses. Descriptive statistics for continuous variables were summarized as mean ± standard deviation or median (25^th^-75^th ^percentile) for variables with a skewed distribution. Categorical variables were summarized using proportions. Baseline characteristics of S319 allele carriers and non-carriers were compared using Welch's modified t test or Chi-Square test as appropriate. Fisher's exact test was used to assess *HNF1A *genotype frequencies. S/S319 and S/G319 genotypes were combined in subsequent analyses due to a small number of S/S319 carriers (n = 1) in the study population. For the purpose of analysis and discussion, individuals with S/S and S/G genotypes will be referred to as "S319 carriers".

Multiple logistic regression analysis was conducted to evaluate the association of the *HNF1A *G319S carrier status with incident type 2 diabetes. The *HNF1A *G319S polymorphism was assessed with adjustment for demographic information (age and sex) in model 1. Subsequent models were additionally adjusted for clinical risk variables, including hypertension, dyslipidemia, hyperglycemia, and insulinemia, that were reported to be associated with incident diabetes in this population [14]: Model 2 was adjusted for model 1 variables in addition to baseline hypertension, triglyceride, HDL cholesterol, and waist circumference (or BMI); model 3 was adjusted for model 2 variables in addition to baseline fasting glucose; and model 4 was adjusted for model 3 variables in addition to baseline HOMA-insulin resistance.

Interactions of *HNF1A *G319S with previously assessed risk variables for incident diabetes [14], including age, BMI, waist circumference, hypertension (yes/no), HDL and LDL cholesterol, triglyceride, fasting insulin, pack-year and active cigarette smoking (yes/no), were tested by adding an interaction term to a model that included the G319S carrier status and the main effect of interest with adjustment for age, sex, hypertension, triglyceride, and waist circumference. Data analyses were performed with the use of SAS software, version 9.2 (SAS Institute, Cary, NC), and with the consideration of two-sided p < 0.05 as statistically significant for all analyses.

## Results

Baseline characteristics of S319 allele carriers and non-carriers are presented in table 1. There were no statistically significant differences between carriers and non-carriers in previously reported clinical risk factors for incident type 2 diabetes and the cigarette smoking status at baseline (p ≥ 0.05) (table 1). Of 481 participants who were free of diabetes at baseline and genotyped for the *HNF1A *G319S polymorphism, 388 (80.7%) G/G319, 92 (19.1%) S/G319, and 1 (0.2%) S/S319 carriers were included in the study. The incidence of type 2 diabetes was higher among S319 allele carriers compared to non-carriers (table 2). The number of baseline active cigarette smokers was not significantly different according to the 10-year incident diabetes status at follow-up (p = 0.68): of 86 individuals with incident diabetes at follow-up, 52 were active cigarette smokers at baseline (60.5%); and of 404 diabetes-free individuals, 254 were active cigarette smokers at baseline (62.9%).

**Table 1:** Baseline characteristics of diabetes-free subjects according to the HNF1A G319S carrier status, the Sandy Lake Health and Diabetes Project (1993-1995)

Characteristic	G319/G319	S319/G319 + S319/S319	p-value
n (%)	388 (80.7)	93 (19.3)	

Age (years)^a^	26.9 ± 13.6	25.1 ± 10.8	0.18

Sex, male^b^	156 (40.2)	47 (50.5)	0.07

Anthropometry^a^			

Body mass index (kg/m^2^)	26.1 ± 5.85	25.9 ± 5.11	0.75

Waist circumference (cm)	96.3 ± 14.7	96.3 ± 12.5	0.95

Blood pressure			

Systolic blood pressure (mmHg)^c^	113.0 (104.0-120.0)	116.5 (106.5-121.0)	0.86

Diastolic blood pressure (mmHg)^a^	65.1 ± 11.9	65.5 ± 11.8	0.77

Hypertension, yes^b,d^	68 (17.3)	13 (14.0)	0.41

Lipid profile			

HDL cholesterol (mmol/l)^a^	1.25 ± 0.28	1.24 ± 0.27	0.77

LDL cholesterol (mmol/l)^a^	2.49 ± 0.74	2.44 ± 0.78	0.56

Triglyceride (mmol/l)^c^	1.16 (0.85-1.61)	1.78 (0.91-1.55)	0.78

Glucose homeostasis			

Fasting glucose (mmol/l)^a^	5.39 ± 0.49	5.33 ± 0.52	0.31

2-hour postload glucose (mmol/l)^a^	5.63 ± 1.75	5.61 ± 1.88	0.93

Fasting insulin (pmol/l)^c^	102.0 (71.0-145.0)	91.5 (57.0-132.0)	0.06

HOMA-Insulin resistance^c^	3.38 (2.24-4.95)	2.85 (1.92-4.27)	0.05

Pack-year^c^	1.25 (0.05-4.80)	1.73 (0.0-6.0)	0.83

Active cigarette smoker, yes^b^	248 (64.3)	55 (59.1)	0.36

a. Mean ± standard deviation and Welch's t test performed.

b. n (%) and Chi-Square test performed.

c. Medians (25^th^-75^th ^percentile) and Welch's t test performed on log transformation.

d. Hypertension is defined as a systolic blood pressure ≥ 130 mmHg or diastolic blood pressure of ≥ 85 mmHg or receiving antihypertensive medication therapy.

Abbreviation: HOMA, homoeostasis model assessment. n of subjects for each characteristic varying slightly due to occasional missing values.

**Table 2:** HNF1A genotype frequencies according to 10-year cumulative incident type 2 diabetes

	G319/G319	S319/G319	S319/S319	S319/G319 + S319/S319
No diabetes	333 (85.8)	64 (69.6)	0 (0)	64 (68.8)

Incident diabetes	55 (14.2)	28 (30.4)	1 (100)	29 (31.2)

p	< 0.001			< 0.001 (vs. G319/G319)

N (%) and Fisher's exact test used.

Multiple regression models were constructed to assess whether the *HNF1A *G319S polymorphism was associated with incident type 2 (table 3). In the first model adjusted for age and sex, the *HNF1A *G319S polymorphism was associated with increased risk for developing type 2 diabetes (odds ratio [OR] 3.24 [95% CI 1.90-5.53]). When the models were further adjusted for clinical risk factors for incident type 2 diabetes (models 2 and 3), the effect size increased (table 3). When model 2 included BMI adjustment instead of waist circumference, the OR was similar to the model including waist circumference (data not shown). In the model adjusted for baseline age, sex, hypertension, triglyceride, waist circumference, HDL cholesterol, and fasting glucose, the OR was 3.93 [95% CI 2.20-7.03]. The addition of HOMA-insulin resistance to this model attenuated the association slightly although it remained statistically significant (3.83 [2.13-6.89]). The associations of *HNF1A *G319S with longitudinal changes in glucose levels as continuous variables were assessed. The *HNF1A *G319S polymorphism was individually associated with 10-year changes in fasting glucose (beta = 0.726, SE = 0.262, p = 0.006) and 2-hour postload glucose (beta = 0.932, SE = 0.469, p = 0.048) after adjusting for age and sex. With adjustment for age, sex, hypertension, triglyceride, waist circumference, and HDL cholesterol, the association of the *HNF1A *polymorphism with 10-year changes in fasting glucose remained significant (beta = 0.645, SE = 0.257, p = 0.01), while the association with 10-year changes in 2-hour postload glucose was attenuated to non-significance (beta = 0.880, SE = 0.470, p = 0.06).

**Table 3:** Multiple logistic regression analysis of the association of the G319S carrier status in HNF1A with the incidence of type 2 diabetes

Covariate adjustment	OR (95% CI)	p
Model 1: age + sex	3.24 (1.90-5.53)	< 0.0001

Model 2: model 1 + hypertension + triglyceride + HDL cholesterol + waist circumference	3.78 (2.13-6.69)	< 0.0001

Model 3: model 2 + fasting glucose	3.93 (2.20-7.03)	< 0.0001

Model 4: model 3 + HOMA-insulin resistance	3.83 (2.13-6.89)	< 0.0001

Abbreviation: OR, odds ratio; CI, confidence interval; HOMA, homoeostasis model assessment. OR and 95% CI per 1-unit change (G319/G319, S319/G319 and S319/S319 corded as 0, 1, and 1).

There were no statistically significant interactions of the *HNF1A *G319S polymorphism with previously reported risk factors of incident diabetes in this population [14] including age, BMI, waist circumference, hypertension (yes/no), HDL and LDL cholesterol, triglyceride, and fasting insulin on the outcome of incident type 2 diabetes after adjustment for age, sex, hypertension, triglyceride, and waist circumference (all p ≥ 0.05) (data not shown), except for fasting insulin (p = 0.03). When participants were stratified by low and high insulin level groups (median = 99 pmol/l), the association of *HNF1A *G319S with incident type 2 diabetes remained significant in both groups although the association in the low insulin group was stronger compared to the high insulin group (OR 6.96 [95% CI 2.73-17.72] and 2.53 [1.14-5.60], respectively).

**Figure 1:**
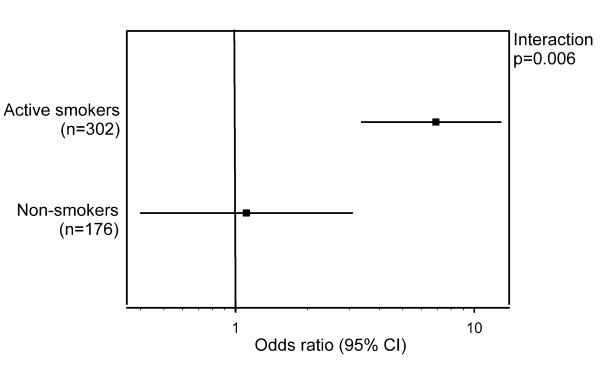
***HNF1A *G319S and incident type 2 diabetes stratified by active cigarette smoking**. Odds ratios are adjusted for age, sex, hypertension, triglyceride, and waist circumference. The distributions of S319 carriers within smokers and non-smokers were 55/303 (18.2%) and 38/176 (21.6%) and were not statistically different (p = 0.36).

An interaction was present between the *HNF1A *G319S carrier status and active cigarette smoking at baseline on the outcome of incident type 2 diabetes (p = 0.006), with adjustment for age, sex, hypertension, triglyceride, and waist circumference. When participants were stratified by active cigarette smoking status at baseline, *HNF1A *G319S was strongly associated with incident type 2 diabetes among active smokers (OR 6.91 [95% CI 3.38-14.12]), while the association was attenuated and became non-significant among non-smokers (1.11 [0.40-3.08]) (figure 1). Interactions of *HNF1A *G319S and active cigarette smoking at baseline on longitudinal changes in glucose levels as continuous variables were assessed. An interaction between *HNF1A *G319S and active cigarette smoking at baseline was not statistically significant on the outcome of 10-year changes in fasting glucose (p = 0.13) with adjustment for age, sex, hypertension, triglyceride, and waist circumference. When participants were stratified by baseline active cigarette smoking status, however, *HNF1A *G319S was strongly associated with fasting glucose changes among active smokers (beta = 1.004, SE = 0.314, p = 0.0016), while the association was not significant among non-smokers (beta = 0.121, SE = 0.448, p = 0.79). An interaction between *HNF1A *G319S and active cigarette smoking at baseline was significant on the outcome of 10-year changes in 2-hour postload glucose (p = 0.027) with adjustment for age, sex, hypertension, triglyceride, and waist circumference. When participants were stratified by baseline active cigarette smoking status, *HNF1A *G319S was strongly associated with changes in 2-hour postload glucose among active smokers (beta = 2.108, SE = 0.611, p = 0.0007), while the association was not significant among non-smokers (beta = -0.122, SE = 0.740, p = 0.87). An interaction between *HNF1A *G319S and baseline pack-years was borderline significant (p = 0.05). Among those who provided both baseline and follow-up smoking behavior information and who were genotyped for *HNF1A *G319S, 142 of 420 (33.8%) participants changed their smoking behavior during 10-year follow-up (84 stopped smoking and 58 started smoking). Therefore, we performed interaction testing between changes in smoking behavior and the *HNF1A *G319S polymorphism and determined that it was not statistically significant (p = 0.50). In addition, the S319 carrier distributions among those who changed their smoking behavior and those who did not were not significantly different (p = 0.30).

## Discussion

We found that the *HNF1A *G319S polymorphism was associated with increased risk of developing type 2 diabetes after adjustment for previously reported baseline clinical risk factors [14]. An interaction between the G319S variant and active cigarette smoking at baseline on the outcome of incident type 2 diabetes was evident after adjustment for age, sex, hypertension, triglyceride, and waist circumference. When participants were stratified by active cigarette smoking status at baseline, S319 was strongly associated with increased risk of developing type 2 diabetes among active smokers, while the association was attenuated to non-significance among non-smokers. Thus, *HNF1A *G319S is associated with the future development of type 2 diabetes in this Aboriginal population, and this association is amplified in the presence of cigarette smoking.

Recently, a type 2 diabetes susceptibility locus near *HNF1A *was identified in large-scale association analysis of predominantly European descent populations [10]. In a prospective cohort study from Scandinavian countries, a functional variant, *HNF1A *I27L, was associated with increased future risk for type 2 diabetes in Scandinavian countries (Hazard Ratio 1.2 [95% CI 1.1-1.3]) [18]. In several studies investigated using case-control designs, however, the positive association between genetic variations in *HNF1A *and type 2 diabetes was not observed [19,20]. Holmkvist et al. [18] argued that case-control studies are less effective at detecting small effects because case and control subjects are often ascertained differently. Hence, prospective cohort studies where all individuals have undergone the same measurements to identify the disease as in the present study and that of Holmkvist et al. [18] would circumvent this issue.

While the *HNF1A *G319S polymorphism demonstrated reduced *in vitro *capacity to transactivate insulin response, this disorder was not as severe as that seen in some nonsense mutations in *HNF1A *that underlie MODY type 3 [9]. Carriers of S319 do not develop MODY; rather in the context of obesity and insulin resistance, carriers appear to have a compromised ability to mount an insulin response, resulting in earlier loss of glycemic control [9]. Recently, the *HNF1A *G319S genomic sequence was reported to give rise to two abnormal transcripts, with altered quantities of the normal splicing products and reduced total *HNF1A *transcript levels [21]. Thus, *HNF1A *G319S might compromise insulin response through at least two mechanisms: a failure of the mutant protein to transactivate insulin response and decreased translation of normal HNF-1α due to abnormal splicing. Since HNF-1α protein plays a key role in β-cell function [4], the reduction in normal HNF-1α activity may compromise β-cell function and further explain the interaction between the *HNF1A *G319S polymorphism and fasting insulin on the outcome of incident type 2 diabetes observed in our study.

The presence of cigarette smoking appears to amplify the predisposition to the development of type 2 diabetes in carriers of *HNF1A *G319S. To our knowledge, there is no previously reported relation between smoking and loss of glycemic control in individuals with other polymorphisms in *HNF1A *or in other MODY genes. A meta-analysis report indicated that active smoking behavior might be generally associated with increased risk for developing type 2 diabetes [22]. In a European cohort study of participants aged 25-74 years, the risk for type 2 diabetes in men increased in a dose-response manner with the increasing number of cigarette consumption [23]. The underlying mechanism whereby cigarette consumption increases diabetes risk is not entirely clear. Nonetheless, smoking has been previously associated with insulin resistance [24,25]. In a non-randomized experimental trial, smoking acutely impaired glucose tolerance and insulin sensitivity, and increased serum LDL cholesterol, triglyceride, blood pressure, and heart rate [24]. In a double-blind, cross-over, placebo-controlled, randomized experimental study, nicotine infusion further aggravated the insulin resistance status among individuals with type 2 diabetes, but not among control subjects without diabetes [25]. Therefore, participants who were likely to have reduced insulin function were more susceptible to the detrimental effects of nicotine. In addition, it was speculated by others that nicotine or other agents in cigarette smoke might directly induce pancreatic injuries [26] and effect insulin secretion by inducing oxidative stress in the pancreas and subsequently inducing loss of β-cells [27]. In the currently study population, we have previously reported that baseline active smoking status was not independently associated with increase risk of incident type 2 diabetes [14]. However, *HNF1A *G319S carriers who were active smokers had increased risk of developing diabetes in the present study. Hence, smokers who carry S319 allele, and therefore are exposed to compromised insulin function [9], are at increased risk of developing diabetes.

Limitations of our study include challenges of conducting investigations in a remote community setting. Specifically, we were unable to collect interim data to analyze the time to the onset of type 2 diabetes. We were also unable to obtain follow-up blood samples from all participants: diabetes outcome assessments of 109 (22.2%) participants were by self-reported clinical diagnosis only. This 22.2% without blood samples might have caused under-reporting of incident diabetes. We did not have a number of cigarettes smoked by former smokers at baseline. Therefore, we excluded 75 former smokers in our smoking dose-response analysis by pack-years (15.6% of total participants who were genotyped). This might have contributed to the attenuated interaction observed between the G319S variant and smoking dose in this study. We also note that our study results, especially the smoking interaction, need to be confirmed in other populations. Nonetheless, the current study was able to retain a high 10-year follow-up rate of 89.1% and was able to investigate the prospective association between the population-specific genetic variation in *HNF1A *and incident type 2 diabetes.

## Conclusions

The population-specific *HNF1A *G319S variant was associated with incident type 2 diabetes in Aboriginal Canadians. Our results support that an environmental variable, cigarette smoking, may influence the development of the diabetes phenotype in the *HNF1A *G319S carriers. This is a particularly important public health concern among Aboriginal populations of Canada who have high prevalence rates of smoking and type 2 diabetes compared to non-Aboriginal populations [1,28]. In future genetic studies, we recommend that potential gene-environment interactions be taken into consideration when investigating the type 2 diabetes as an outcome.

## Competing interests

The authors declare that they have no competing interests.

## Authors' contributions

All of authors read and approved the final manuscript and contributed in revising the manuscript critically for important intellectual content. SHL contributed to the statistical analysis and interpretation of the data and drafted the manuscript. RAH and HC contributed to the genetic analysis and interpretation of the data. MM contributed to the acquisition of data. RAH, PWH, SBH, JG, RR, BZ and AJH contributed to the conception and design of the study.

## Pre-publication history

The pre-publication history for this paper can be accessed here:

http://www.biomedcentral.com/1471-2350/12/1/prepub
